# From startup to shutdown: the dramatic rise and fall of the first at-home combo test for flu and COVID-19

**DOI:** 10.1039/d5lc00305a

**Published:** 2025-09-25

**Authors:** Morgan N. Greenleaf, Gregory L. Damhorst, Eric M. Vogel, Greg S. Martin, Wilbur A. Lam

**Affiliations:** a The Atlanta Center for Microsystems-Engineered Point-of-Care Technologies Atlanta Georgia USA; b Division of Infectious Diseases, Department of Medicine, Emory University School of Medicine USA; c Division of Pulmonary, Allergy, Critical Care, and Sleep Medicine, Department of Medicine, Emory University School of Medicine USA; d Division of Pediatric Hematology/Oncology, Department of Pediatrics, Aflac Cancer Center and Blood Disorders Service of Children's Healthcare of Atlanta, Emory University School of Medicine Atlanta GA 30322 USA; e Wallace H. Coulter Department of Biomedical Engineering, Emory University and Georgia Institute of Technology, Emory University and Georgia Institute of Technology USA Wilbur.lam@emory.edu; f Georgia Clinical and Translational Science Alliance USA; g School of Materials Science and Engineering, Georgia Institute of Technology Atlanta GA 30332 USA

## Abstract

This article explores the development and commercialization of Lucira Health's innovative at-home molecular diagnostic test, which detects influenza A or B and SARS-CoV-2. Launched amidst the urgent demand for accessible testing solutions, Lucira's product represented a significant breakthrough, becoming the first over-the-counter combination test authorized by the US Food and Drug Administration (FDA). The narrative tracks Lucira's journey from its origins in microfluidics at the University of California-Berkeley, through development challenges, business success and failure. It also contrasts the distinct motivations and technical challenges of pre-pandemic *versus* pandemic era diagnostics, emphasizing test-to-treat strategies *versus* rapid results for containment. Despite early successes, Lucira faced insurmountable regulatory and financial hurdles, culminating in bankruptcy just days before FDA authorization. The case offers critical insights into diagnostics product development, regulatory navigation, product diversification, and strategic risk management in push towards home and point of care diagnostics.

## Abstract 2

Lucira health|innovation, rise, and fall.

## Graphical abstract

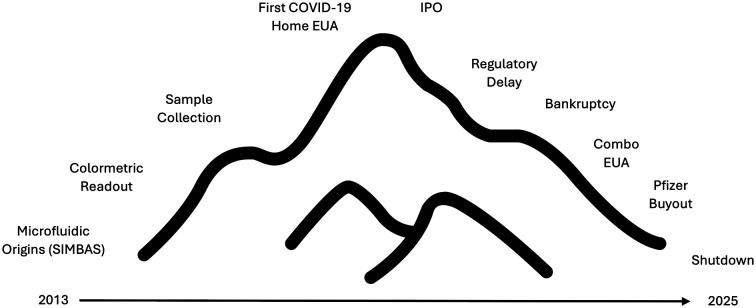

The NIH-funded Atlanta Center for Microsystems Engineering POC Technologies (ACME POCT) is focused on microsystems-based and microfluidics-based diagnostics. The center's experience with the Rapid Acceleration of Diagnostics (RADx) program to speed the development, validation, and commercialization of innovative SARS-CoV-2 point-of-care and home based tests has brought the ACME POCT team into contact with amazing leaders of diagnostic technology companies. This experience has taught the team tremendously about the business and technology of microfluidics commercialization. As such, we aim to disseminate this knowledge to our microfluidics community by publishing a series of “lessons learned” case studies focused on the technical, clinical validation, regulatory, and commercialization lessons that led to company success or company failure. Our third case in this series discusses the development and commercialization of the Lucira Covid and Covid/flu combination home tests. ACME POCT is co-directed by Wilbur Lam, MD, PhD, Greg Martin, MD, MSc and Eric Vogel, PhD.

## Introduction|the first combo test for home use

On February 24th, 2023, the US Food and Drug Administration (FDA) issued the first Emergency Use Authorization^1^ for a combination influenza A, influenza B, and SARS-CoV-2 test for home use. Not only was this the first authorized combo test for influenza and SARS-CoV-2 by the agency, but it was the first over the counter (OTC) authorization for an influenza test in the United States. The excitement was tempered by one simple fact. The company producing the test, Lucira Health Inc. (Lucira) had just declared bankruptcy two days earlier on February 22nd 2023.^2^

What follows is an account of the events that led to February 2023 in three acts: act one will cover the creation of the Lucira test product and company, act two will discuss the difference between pre-pandemic and pandemic era testing objectives, act three will cover the regulatory approval of the combo test, and finally we end with an epilogue and discussion. Throughout, we highlight the business strategy of diagnostics and provide advice for future diagnostic developers.

## Act one|molecular testing for the home

**Fig. 1:**
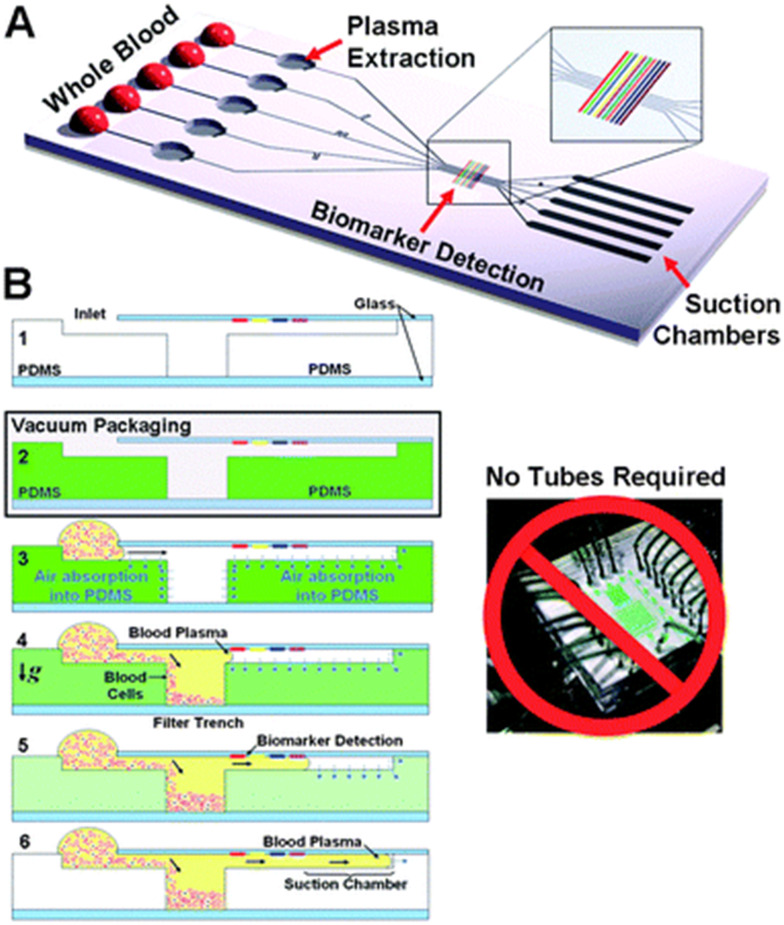
Self-priming, self-contained, tether-free SIMBAS (A) integrates volume metering, plasma separation from whole-blood, multiple biomarker detection, and suction chambers for fluid propulsion. (B) (1) Cross section of device operation: (2) storage in low pressure, *e.g.* vacuum package; (3) within 2 min of removing the device from vacuum conditions and placing a 5 mL whole-blood sample on the inlet, degas-driven flow propels the sample into the device; (4) as the whole-blood passes over the filter trench, blood cells sediment gravitationally and are filtered while plasma flows into the channel; (5) plasma-based proteins are detected as the plasma flows across the biomarker detection zone; (6) suction chamber regulates the total volume of plasma analyzed and stops the flow before the trench filters are overfilled. Reproduced from ref. 3 with permission from the Royal Society of Chemistry, Copyright 2011.

Lucira was started in 2013 as Diassess, Inc. by Drs. Debkishore Mitra, John Waldeisen, and Ivan Dimov. The cofounders met at the University of California Berkley while working on microfluidics in the Department of Bioengineering under the supervision of Professor Luke Lee. The core microfluidic technology was developed in Professor Lee's group based on the self-powered integrated microfluidic blood analysis system (SIMBAS). SIMBAS utilized degas-driven fluid flow by leveraging the gas permeability of PDMS, allowing passive fluid propulsion when exposed to atmospheric pressure. The system integrates blood plasma separation using a trench-based filtration mechanism that traps red and white blood cells while allowing plasma to flow into a biomarker detection zone. Additional details can be found in the original manuscript (Fig. 1).^3^

The goal of the company was to bring molecular level sensitivity to the home in a disposable form factor, first starting with diagnosis of common sexually-transmitted infections (STIs) caused by the bacteria *Chlamydia trachomatis* and *Neisseria gonorrhoeae* (CT/NG). The company's goals were specific: molecular level sensitivity was important to accurately detect the target pathogens but also because the “test to treat” paradigm was meant to facilitate early detection (at lower pathogen loads) so that treatment could be sought by the patient, curbing the spread of STIs. Further, the company was focused on disposable tests because they did not believe infectious disease diagnosis would ever be a “lifestyle product” in which a dock/cassette model would be better suited. Thus, the company was guided by these product requirements: a disposable and highly sensitive molecular test for use in the home.

## Business concept|Blue Ocean Strategy

“Competing in overcrowded industries is no way to sustain high performance. The real opportunity is to create blue oceans of uncontested market space”.

Blue Ocean Strategy: How to Create Uncontested Market Space and Make the Competition Irrelevant by W. Chan Kim and Renee Mauborgne.^4^

Blue Ocean Strategy emphasizes creating new, untapped markets (“blue oceans”) rather than competing in crowded, established industries (“red oceans”). The strategy aims to generate demand by offering innovative products or services that redefine the market, often through differentiation (*e.g.* molecular sensitivity at home). While perhaps unintentional, Lucira focused on two blue oceans, the molecular home testing market and the home market for infectious diseases, neither of which were crowded with competitors yet.

### Technical product development

The company broke the development of the product into three phases, 1) technical innovation to create a disposable molecular test, 2) design of sample collection, and 3) usability refinements.

Technical innovations required to create the Lucira home test included microfluidic advancements and optoelectronic development and integration. As the technology moved beyond the proof of concept stage, one key change was moving from fluorescent to colorimetric readout.^5^ This reduced device cost and allowed the product to be disposable. In addition, while originally the team designed the microfluidics using polydimethylsiloxane (PDMS) which is ubiquitous in microfluidics development, the company moved away from this material because of its lack of reproducibility at scale. PDMS requires a slow manufacturing process with many steps that are hard to replicate exactly. Moving away from PDMS required the team to replace the degas-driven fluid flow from the original product with a pressure driven flow method with electrically driven pumps (Table 1).

**Table 1:** Selected Lucira health patents covering product research and development

First filing date	Patent title	Technical innovation
2014-04-24	Colorimetric detection of nucleic acid amplification	Colorimetric readout
2016-03-14	Devices and methods for biological assay sample preparation and delivery	Pressure driven fluid flow
2016-03-14	Devices and methods for modifying optical properties	Optoelectronic development and integration

The second phase of development was focused on sample collection. After initially focusing on CT/NG, the team decided this regulatory path was too difficult to bring a product to market. The team pivoted to Influenza. The standard nasopharyngeal (NP) swab is difficult for an untrained individual to collect, and therefore not appropriate for home use. The team performed a study comparing NP swabs, midturbinate (MT) swabs, and anterior nasal swabs for accuracy and discomfort^6^ and found that MT swab sensitivity for detecting influenza (A or B) was 98% (95% CI 94.25% to 99.65%) compared to NP with significantly reduced discomfort.

In phase three, the company focused on usability and human factors. The company hired a consumer product expert and conducted hundreds of usability studies. One key change was moving from an LCD display to a very simple LED based display which was easier for users to interpret and further reduced the product's cost.

**Fig. 2:**
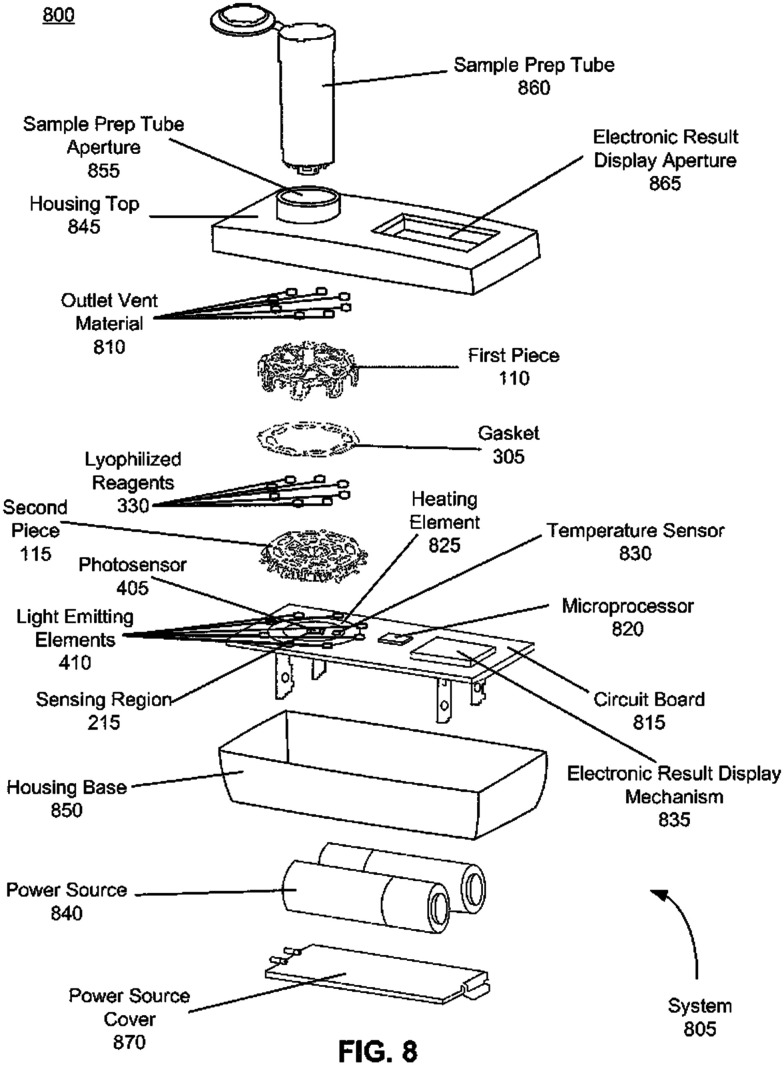
Schematic of Lucira device patent. Reproduced from ref. 7 with permission Frank Myers, Copyright 2020.

The final product platform which can support multiple RNA targets utilizes RT-LAMP to detect viral RNA in an all-in-one test kit. The product creates an amplification reaction that induces a color change of halochromic agents which is detected by optoelectronic components and analyzed by an onboard microprocessor. Results are displayed to the user *via* LED in as little as 11 minutes for a positive and up to 30 minutes for negative (Fig. 2).

Throughout the development of the Lucira test, the company raised substantial capital (>$284 M) from both dilutive and non-dilutive sources (Table 2).

**Table 2:** Investment funding received by the company

Year	Type	Direct	Indirect	Total	Dilutive/non-dilutive
2012	NIH	$148 000.00		$148 000.00	Non-dilutive
2014	SBIR/STTR	$295 568.00		$295 568.00	Non-dilutive
2015	SBIR/STTR	$295 568.00		$295 568.00	Non-dilutive
2015	Y Combinator	$120 000.00		$120 000.00	Dilutive
2015	Series A	$12 770 000.00		$12 770 000.00	Dilutive
2016	SBIR/STTR	$656 897.00		$656 897.00	Non-dilutive
2017	SBIR/STTR	$651 558.00		$651 558.00	Non-dilutive
2017	SBIR/STTR	$668 896.00		$668 896.00	Non-dilutive
2018	SBIR/STTR	$722 035.00		$722 035.00	Non-dilutive
2019	Contract	$21 900 000.00		$21 900 000.00	Non-dilutive
2019	Series B	$32 500 000.00		$32 500 000.00	Dilutive
2020	SBIR/STTR	$729 647.00	$269 581.00	$999 228.00	Non-dilutive
2020	Series C	$58 700 000.00		$58 700 000.00	Dilutive
2021	SBIR/STTR	$738 937.00	$252 406.00	$991 343.00	Non-dilutive
2021	IPO	$153 000 000.00		$153 000 000.00	Dilutive
2022	SBIR/STTR	$730 587.00	$202 406.00	$932 993.00	Non-dilutive
Total		$284 627 693.00			

## Act two|pre-pandemic *vs.* pandemic era

The two key product requirements guiding the development of the Lucira Flu test were 1) a highly sensitive molecular test and 2) a disposable test. The first requirement arises from the motivations for influenza testing in the pre-covid pandemic world, which were to make treatment decisions, conduct infection control and prevention measures in clinical settings, and for public health surveillance.^8^ However, clinical management – making decisions about antiviral treatment at outpatient clinics (urgent care and primary care) and hospital emergency departments – turned out to be what drove testing volume.

### Pre-pandemic

Several point of care influenza products existed during the development of the Lucira test including rapid antigen tests, rapid molecular tests, and enhanced antigen tests with greater sensitivity. These tests were available in point of care settings (so called CLIA-waived settings) such as outpatient medical facilities and retail pharmacies, where the tests facilitated the test to treat paradigm, but were not authorized for use over-the-counter or in the home. Antiviral medications such as oseltamivir (Tamiflu), zanamivir (Relenza), peramivir (Rapivab), baloxavir marboxil (Xofluza) are typically indicated for use within 48 hours of symptom onset, thus a diagnostic that can detect influenza virus within the first two days of illness course is required (Table 3).

**Table 3:** Influenza medication guidelines

Influenza medication	Administration guidelines
Oseltamivir	Therapy should begin within 48 hours from onset of signs or symptoms. Not recommended for treatment of severe or progressive influenza or patients with disease severe enough to warrant hospitalization, due to lack of data^9^
Zanamivir	Therapy should begin within 48 hours from onset of signs or symptoms. Not recommended for treatment of severe or progressive influenza or patients with disease severe enough to warrant hospitalization, due to lack of data^9^
Peramivir	600 mg as a single dose; initiate within 2 days of onset of symptoms of influenza. Due to insufficient data on use of peramivir for treatment of hospitalized patients with influenza, only consider for patients who cannot tolerate or absorb oral or enterically-administered oseltamivir due to gastric stasis, malabsorption, or GI bleeding^9^
Baloxavir marboxil	Single dose within 48 hours of onset of influenza symptoms^9^

In addition to guiding antiviral therapy, influenza diagnostics were used to prevent unnecessary antibiotic or antiviral treatment, and outbreak control in high-risk populations (nursing homes, hospitals).

### Point of care influenza diagnostics available pre-covid

**Fig. 3:**
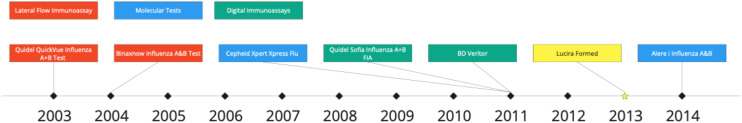
Existing influenza POC diagnostics.

Rapid influenza diagnostic tests (RIDTs) were available pre-covid such as Binaxnow Influenza A&B Test (Authorized 2004) and the Quidel QuickVue Influenza A+B Test (Authorized 2003). Both are lateral flow immunoassay designed for the rapid, qualitative detection of influenza A and B antigens. In addition to antigen tests, rapid molecular assays including the Cepheid Xpert Xpress Flu (Authorized 2011) and Alere i Influenza A&B (Authorized 2014) were available as instrument/consumable diagnostic options. Finally digital Immunoassays (DIAs) and enhanced RIDTs such as the Quidel Sofia Influenza A+B FIA (Authorized 2011) and BD Veritor™ System for Rapid Detection of Flu A+B (Authorized 2011) improved on the sensitivity and specificity of RIDTs. In high complexity settings (*e.g.* hospitals), reverse transcriptase polymerase chain reaction (RT-PCR) was commonly used due to superior sensitivity and specificity (Fig. 3).

### Pandemic-era

SARS-CoV-2 changed the motivations for infectious disease testing of influenza-like illness from pre-pandemic test to treat, to pandemic era stop the spread. Now, instead of focusing on an individual's desire for treatment of their own disease, the primary testing motivation was symptom confirmation and diagnosis coupled with containing the spread of disease by identifying and isolating infected individuals, thereby protecting individuals more vulnerable to COVID-19. This is euphemistically referred as “don't get grandma sick”. This shifting paradigm changed test requirements. Instead of highly sensitive tests that could detect low levels of virus but are only accessed in healthcare settings, pandemic-era requirements created a role for highly accessible, low cost, rapid results tests, even if at a loss of sensitivity. See Table 4 which highlights the Lucira COVID-19 sensitivity of 94.1% PPA compared to rapid antigen lateral flow immunoassays BinaxNOW COVID-19 (81.6% PPA) and QuickVue COVID-19 (83.5% PPA).

**Table 4:** Comparison of performance for COVID-19 assays that previously were authorized for influenza

Test	Analytical performance		Clinical performance					
	Sensitivity	Note	PPA	95% CI		NPA	95% CI	
Lucira COVID-19 All-In-One Test Kit	2700	GE per swab	94.1	85.5	98.4	98	89.4	99.9
BinaxNOW™ COVID-19 Ag Card Home Test	140.6	TCID50 per mL	81.6	73.7	88	98.3	95.6	99.5
QuickVue At-Home OTC COVID-19 Test	19 100	TCID50 per mL	83.5	74.9	89.6	99.2	97.2	99.8
Xpert Xpress SARS-CoV-2	0.02	PFU mL^−1^	97.8	88.4	99.6	95.6	85.2	98.8
ID NOW COVID-19 2.0	500	Copies per swab	93.3	89.5	96.1	98.5	97.2	99.3
Sofia SARS Antigen FIA	113	TCID50 per mL	96.7	83.3	99.4	100	97.9	100
BD Veritor™ System for Rapid Detection of SARS-CoV-2	140	TCID50 per mL	84	67	93	100	98	100

In this environment, antigen lateral flow assays had distinct advantages. The tests are low cost to manufacture and distribute at massive scale and relatively easy to operate by the public in a home environment. In addition, the government provided billions of tests free to the public through the public mail system.

In contrast to behavioral motivations in pre-pandemic time (“do I need treatment?”), now the focus is on, “am I negative?” Consumers seeking negative test results for travel, to attend events, or see relatives prioritized affordable and accessible tests, which generally were highly specific, even if at the expense of sensitivity. In this wartime environment, the highly sensitive but more expensive Lucira COVID-19 test was popular with specific adopter groups including the highly educated and communities with specific risks (*e.g.* weakened immune system, cancer treatment), but not the broader public. Lucira had designed and developed a product for a pre-pandemic world – testing for influenza with different diagnostic requirements – than what a pandemic era COVID-19 diagnostic required.

## Act three|business strategy for regulated single product companies

Years of work by the Lucira team developing the flu home product enabled a quick pivot during the pandemic to work on SARs-CoV-2. In November 2020, the company gained the very first emergency use authorization for self-testing at home with their Lucira COVID-19 All-In-One Test Kit.^10^ Sales boomed and peaked in the first quarter of 2022, selling $90.5 M worth of tests.^11^ However, by the second quarter of 2022, Lucira sold only $26.1 M in tests, a precipitous 71.2% decrease in sales.^12^ The company knew they were in trouble but were already hard at work on the next product, a combo SARS-CoV-2 and Influenza A&B test. Work to validate the test had begun the previous year but was hampered in their clinical trial by a scarcity of Influenza B circulating in the community, an average of 2.3% of samples in the last 13 weeks of the year. Lucira was optimistic, however, as a home use combo test was of high interest to retailers and conversations with a large pharmacy chain concluded with a contract for a large volume purchase of the future combo test. With fingers crossed, the company submitted for OTC authorization to both the FDA and Health Canada in May 2022.

The company's business plan called for a spring submission to the FDA, ramp up manufacturing to meet the contract volume commitments through the summer, then gain authorization in the fall in time to meet the demand of the winter Influenza season in the United States. This plan appeared to be working as their contract manufacturer Jabil ramped up production in the Dominican Republic and Health Canada authorized the combo test on August 11th 2022 however the Canadian market is small compared to the United States.^13^

In September 2022, Lucira heard back from the FDA and it was not good news. The agency was concerned about the toxicity of a chemical used in the buffer solution (guanidine hydrochloride) which could be ingested by users. During the development of the COVID test, Lucira worked collaboratively with the FDA and received clear and straightforward guidance. However, guidance on the combination product was less clear. Thinking the FDA required data to demonstrate low risk, the company undertook additional toxicology studies at great expense. But in fact, the agency required the chemical to be removed from the buffer completely.

By this time, the company was under tremendous financial strain with significant perishable inventory building up in storage that could not be sold. The company raced to redesign the assay, moving the chemical from the buffer solution into the sealed portion of the assay, removing the risk of ingestion. In a stepwise fashion, the agency authorized the Lucira combo test for CLIA waived sites (POC) on November 22 2022; however, this did not help the company because they were focused on the OTC market and had aligned all their marketing, sales, and distribution towards this effort.

On February 24th 2023, the FDA authorized the Lucira combo test for at-home use just two days after the company declared bankruptcy. While announcing the EUA for the combo test,^14^ Lucira President and CEO Erik Engelson cited “what became a protracted authorization cycle time” and lack of clarity when authorization would come “despite working closely with FDA”. In a rare public statement,^15^ the FDA's director of the Center for Devices and Radiological Health pushed back, citing both “risk to consumers due to a toxic substance identified in one of the test components” and a lack of clinical data in original submission. “In addition, the EUA request included only 9 positive influenza A clinical samples – an amount we found was insufficient to adequately determine test performance and support authorization by the FDA”.

## Business concept|product diversification

Diagnostic startups must, by necessity, focus relentlessly on getting a single product to market. This is required because of the expense to achieve marketing approval in the highly regulated medical device market and successfully launch a product commercially. If the company does not focus, cash from investors may be exhausted before a product reaches market and brings in revenue.

However, focusing on a single product has high risks, as shown by the Lucira case. Lucira focused solely on the home market for highly sensitive tests for covid and flu. When their product launch ran into delays, the company had no alternatives to raise revenue and were forced into bankruptcy.

## Epilogue|test to treat through Lucira by Pfizer

On April 20th 2023, Pfizer announced they had purchased Lucira through bankruptcy auction for $36.4 M, just 6% of its IPO valuation of $628.4 M in early 2021. The purchase brought the company full circle to the pre-pandemic environment where treatment of individuals within 48 hours has returned to motivate testing behavior. Pfizer, the maker of the most popular COVID-19 antiviral Paxlovid sought to connect home diagnostics with prescriptions for this medication. The acquisition was part of a larger strategic push to boost sales of Paxlovid due to a predicted decrease in sales of 58% in 2023. In addition to the Lucira purchase, Pfizer struck marketing and co-branding deals with Cue health and Roche to connect a positive test result with the risk of severe illness from COVID-19. After having rebranded the product and received updated FDA authorization, Pfizer decided to shutter its diagnostics division including the Lucira by Pfizer product, which finally ended the product's sale in March 2025, 12 years after the company started.^16^ The authors can only assume Pfizer decided to focus on its core pharmaceutical business.

### The role of timing & luck

The product lifecycle or PLC is a common business framework to think about the stage of a market and a product within that market.^17^ The four stages of the product lifecycle are introduction (sometimes called market development), growth, maturity, and decline. Lucira's home use molecular diagnostic fell squarely within the introduction stage which is the most difficult stage of the product lifecycle when products and their companies often fail. Companies often choose whether to focus on a “first mover advantage” and take on additional risk of failure or the company may choose a less risky path as a “fast follower” if the market is proven lucrative by an initial entrant. Lucira's strategy of moving first into the blue ocean of home molecular diagnostics without a proven consumer pay market increased the risk of failure. Beyond consumer demand risk, the concepts of “first mover” and “fast follower” are codified in the FDA regulatory structure of *De Novo* applications and 510k applications. 510k applications, are by definition products that follow a *De Novo* application, require less clinical and analytical data, and are deemed equivalent to the first *De Novo* authorization. Lucira's subsequent demise appeared to show the industry that a home molecular test for respiratory infections was not in fact demanded by the market outside of a pandemic era. However, some companies are moving towards home molecular sexually transmitted infection (STI) tests as demonstrated by Visby Medical's successful FDA authorization in March 2025 of its home test for Chlamydia, Gonorrhea and Trichomoniasis.^18^

**Fig. 4:**
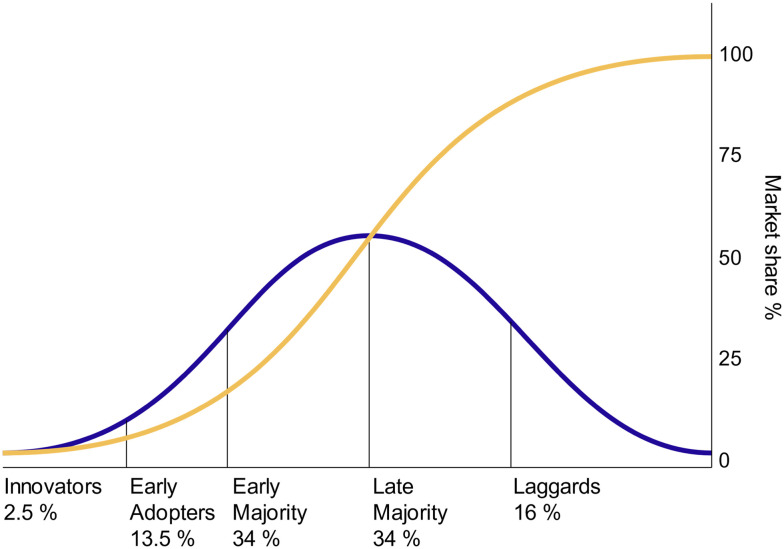
With successive groups of consumers adopting the new technology (shown in blue), the product's market share (shown in yellow) will eventually reach 100%. Adopter Categories. Rogers Everett, Public domain, *via* Wikimedia Commons.

Another lens to view the role of luck and timing in Lurica's success and failure is through diffusion of innovations theory popularized by Everett Rogers.^19^ Diffusion of Innovation Theory examines how ideas and technologies are spread through people and markets and contains four categories that influence the spread: the innovation, communication channels, time, and the social system. The people, or adopters, of the technology are categorized into innovators, early adopters, early majority, late majority, and laggards (Fig. 4).

Lucira succeeded in spreading their technology to innovators and early adopters, but were not able to cross into the early majority. Crossing into the early majority is sometimes referred to as “crossing the chasm”^20^ and requires pivoting the marketing and positioning of the product for a set of adopters with different priorities than the existing adopters. An analysis of the factors that influence adopters decisions shows why (Table 5):

**Table 5:** Diffusion of innovation factors impacting the luck and timing of Lucira success/failure

Factor	Application to Lucira adoption
Compatibility	Before the pandemic, testing for respiratory diseases at home was very uncommon and compatible with consumer habits, insurance/payer markets, or even clinical workflows. During the pandemic, respiratory disease testing at home become much more compatible
Trialability	Consumers must buy the kit making trialability low. Given the relatively higher cost of the Lucira kit, this reduced trialability compared to lower cost alternatives
Relative advantage	The Lucira test had clear relative advantage in speed to result (compared to lab based PCR) and sensitivity (compared to home antigen tests). These relative advantages were most pronounced during the pandemic and reduced in the post-pandemic era
Observability	Home testing is a low observability activity
Simplicity/complexity	Relatively higher compared to antigen tests

Lucira was able to build a market in innovators and early adaptors such as high-risk (*e.g.* immunocompromised) individual and technology or science enthusiasts but the jump to the early majority market was not possible as the pandemic waned and the relative advantage of the technology was reduced.

## Conclusion

When Lucira brought their molecular COVID-19 diagnostic to market and held a successful initial public offering (IPO), it offered a beaming example of the promise and success of academic microfluidics translating into real world products. However, the company's rise was matched with an equal fall into bankruptcy. The rise and fall of Lucira highlights that microfluidic diagnostic products need more than a great technical innovation, they also need great timing matched with great planning and execution for inevitable regulatory and business hurdles.

## Conflicts of interest

There are no conflicts to declare.

## Data Availability

Transcripts of interviews are available upon request.
